# The validity of the Ligs digital arthrometer at different loads to evaluate complete ACL ruptures

**DOI:** 10.3389/fbioe.2023.1049100

**Published:** 2023-03-14

**Authors:** Junqiao Li, Jiexi Tang, Lei Yao, Weili Fu, Qian Deng, Yan Xiong, Jian Li

**Affiliations:** Department of Orthopedics, Orthopedic Research Institute, West China Hospital, Sichuan University, Chengdu, China

**Keywords:** anterior cruciate ligament, knee laxity, quantitative assessment, Ligs digital arthrometer, diagnostic value

## Abstract

**Objective:** The Ligs Digital Arthrometer is a recently launched versatile arthrometer that can be used for the quantitative assessment of knee and ankle joint laxity. This study aimed to evaluate the validity of the Ligs Digital Arthrometer for the diagnosis of complete anterior cruciate ligament (ACL) ruptures at different loads.

**Materials and Methods:** From March 2020 to February 2021, we included 114 normal subjects and 132 subjects diagnosed with complete ACL ruptures by magnetic resonance imaging (MRI) and eventually confirmed by arthroscopy in the study. Anterior knee laxity was independently measured by the same physical therapist using the Ligs Digital Arthrometer. Recorded anterior knee laxity and calculated the side-to-side difference (SSD) at 30, 60, 90, 120, and 150 N loads, respectively. The receiver operating characteristic (ROC) curve was used to determine the optimal laxity threshold, and the diagnostic value was evaluated by the area under the curve (AUC).

**Results:** The demographic data of the subjects were comparable between the two groups (*p* > 0.05). The mean values of anterior knee laxity measured by the Ligs Digital Arthrometer between the complete ACL ruptures group and the control group were significantly different at 30, 60, 90, 120, and 150 N loads (*p* < 0.001 for all). According to the results of ROC curve analysis, the optimal laxity threshold for the diagnosis of complete ACL ruptures was 1.1 mm SSD (Se = 66.7%, Sp = 69.3%) at 30 N, 1.3 mm (Se = 74.2%, Sp = 82.5%) at 60 N, 1.6 mm (Se = 79.5%, Sp = 94.7%) at 90 N, 1.9 mm (Se = 84.1%, Sp = 92.1%) at 120 N and 2.1 mm (Se = 85.6%, Sp = 91.2%) at 150 N. The AUC order at different loads from high to low was 150 N (0.948 [0.923–0.973])>120 N (0.933 [0.903–0.963])>90 N (0.902 [0.862–0.943])>60 N (0.846 [0.799–0.893])>30 N (0.720 [0.657–0.783]).

**Conclusion:** The Ligs Digital Arthrometer proved to be of high diagnostic value in complete ACL ruptures at 90 N, 120 N, and 150 N loads. The diagnostic value improved with the increase of load in a certain range. Based on the results of this study, as a portable, digital and versatile new arthrometer, the Ligs Digital Arthrometer was a valid and promising tool for diagnosing complete ACL ruptures.

## 1 Introduction

Anterior cruciate ligament (ACL) ruptures are the most common type of knee injury with more than 250,000 ACL ruptures annually in the United States. Up to 65% of patients with ACL ruptures undergo anterior cruciate ligament reconstruction (ACLR) surgery; however, some patients with ACL ruptures can be treated with conservative therapy and rehabilitation training instead of operative therapy (Musahl and Karlsson, 2019; Kakavas et al., 2021). Considering this diversity of treatments, clinical diagnosis and evaluation after ACL ruptures are of great significance for selecting proper treatment. Therefore, the objective quantification assessment of anterior knee laxity using an arthrometer is necessary for ACL ruptures (Rohman and Macalena, 2016; Ryu et al., 2018).

Current arthrometers mainly include the KT-1000/KT-2000, Rolimeter and GNRB. Although the KT-1000/KT-2000 (MEDmetric Corp, San Diego, United States) is the most widely used arthrometer with generally supportive diagnostic validity, it can only provide specific loads to diagnose ACL ruptures. Additionally, precision is relatively low at 1 mm and a reading error exists because an artificial reading is required (Robert et al., 2009; Rohman and Macalena, 2016; Saravia et al., 2020). The Rolimeter (Aircast Europa, Neubeuern, Germany) is a device that requires the examiner to record anterior knee laxity by performing the Lachman test at maximal manual force, but this device has several drawbacks including an uncontrolled load and poor repeatability (Balasch et al., 1999; Papandreou et al., 2005; Rohman and Macalena, 2016). The GNRB (Genourob, Laval, France) is a computerized arthrometer with high precision that can provide a constant thrust load and automatically record the corresponding knee laxity, and has been proven to have good validity by several studies (Robert, et al., 2009; Klouche et al., 2015; Rohman and Macalena, 2016; Ryu, et al., 2018; Saravia, et al., 2020). Moreover, as a stress radiography system, the Telos device (GmbH, Hungen, Germany) can directly evaluate anterior tibial translation with high repeatability and no interference with soft tissue. However, it requires the operator to measure radiographs, which is expensive and includes a certain amount of radiation exposure for patients (Jorn et al., 1998; Beldame et al., 2012; Lefevre et al., 2014; Bouguennec et al., 2015; Rohman and Macalena, 2016; Ryu, et al., 2018).

The recently launched Ligs Digital Arthrometer (Innomotion Inc., Shanghai, China) is a versatile arthrometer with portable, digital and radiation-free characteristics that can be used for the quantitative assessment of knee and ankle joint laxity. It has a built-in pressure sensor with an accuracy of 1 N and can provide continuous or constant loads as required. During the measurement, the push rod of the Ligs Digital Arthrometer applies vertical load on the tibia from the rear of the lower leg parallel to the tibial tubercle. The relative displacement is automatically collected in real time by a displacement sensor on the top of the push rod, with a precision of 0.1 mm and a sampling frequency of 30 Hz. Collected data are processed and analysed using the built-in PC system to obtain a force-displacement curve displayed on the screen. In addition, it can perform simultaneous stress radiography with the assistance of radiological equipment when necessary.

At present, the Ligs Digital Arthrometer has been reported in the literature for the quantitative assessment of chronic ankle instability and knee laxity after ACLR (Chen et al., 2022; Zhou et al., 2022). However, there are no studies concerning the application of the Ligs Digital Arthrometer in the diagnosis of ACL ruptures. Therefore, the purpose of this study was to evaluate the validity of the Lig Digital Arthrometer for the diagnosis of complete ACL ruptures at different loads using arthroscopic assessment as the reference standard.

## 2 Materials and methods

A cross-sectional study from March 2020 to February 2021 was conducted. This study was approved by the Ethics Committee on Biomedical Research, West China Hospital of Sichuan University (No. 2016–99 11/1/2016). All subjects understood the purpose and significance of this study and signed an informed consent form.

### 2.1 Inclusion and exclusion criteria

Subjects in the experimental group included the patients in our hospital between 18 and 60 years of age, who were diagnosed with complete ACL ruptures by magnetic resonance imaging (MRI) and eventually confirmed by arthroscopy. The exclusion criteria were as follows: 1) ACL avulsion fracture; 2) concomitant PCL injury; 3) previous surgery and primary disease in the involved knee; 4) previous injury, surgery and primary disease in the contralateral knee; or 5) refusal to participate in the research. Meanwhile, normal people aged between 18 and 60 years were recruited as the control group. The exclusion criteria were as follows: 1) previous injury, surgery and primary disease in either knee; or 2) refusal to participate in the research.

### 2.2 Measurement of anterior knee laxity

**FIGURE 1:**
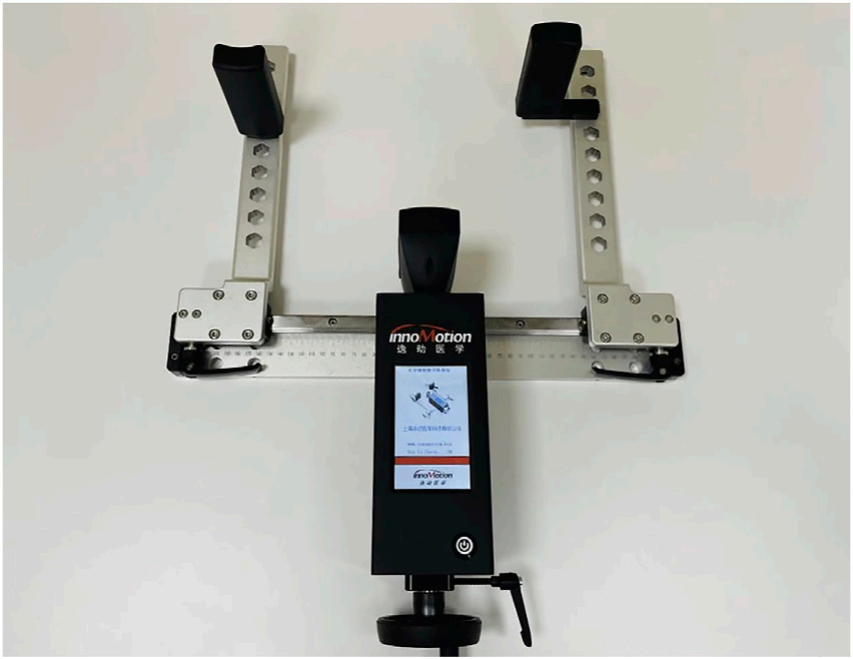
The presentation of the Ligs Digital Arthrometer.

Anterior knee laxity was independently measured by the same physical therapist after strict training using the Ligs Digital Arthrometer (Figure 1). In the experimental group, the healthy knee was measured followed by the involved knee. In the control group, the operator first measured the left knee followed by the right knee.

**FIGURE 2:**
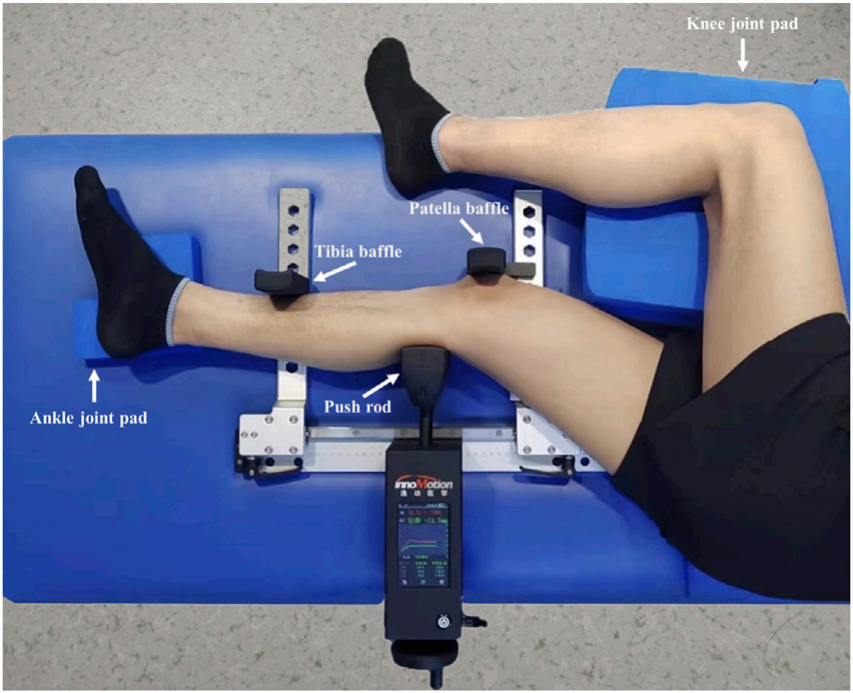
The measurement of anterior knee laxity by the Ligs Digital Arthrometer.

The subject was placed in the standard lateral decubitus position with the involved lower limb on the Ligs Digital Arthrometer. The involved knee flexed at 30° with neutral rotation, and a pad was used to support the ankle joint to keep the tibia horizontal without internal and external rotation. Meanwhile, the contralateral lower limb was bent and placed in front of the body, and the knee joint was supported with a pad to keep it in a relaxed state. The operator aimed the centre of the push rod at the level of the tibial tubercle from the rear of the lower leg, then adjusted the upper bracket to make the patella close to the groove of the patella baffle, and then moved the lower bracket to the end to fix the lower tibia using the tibia baffle (Figure 2).

Before each measurement, the operator reset the arthrometer and instructed the patient to relax the muscles of the lower limbs. During the measurement, a forward load ranging from 0 to 150 N was manually applied to the tibia at a constant speed. If the subject experienced any significant discomfort, the measurement procedure was stopped immediately or at any time upon the subject’s request. The contralateral knee joint was measured by the same method.

**FIGURE 3:**
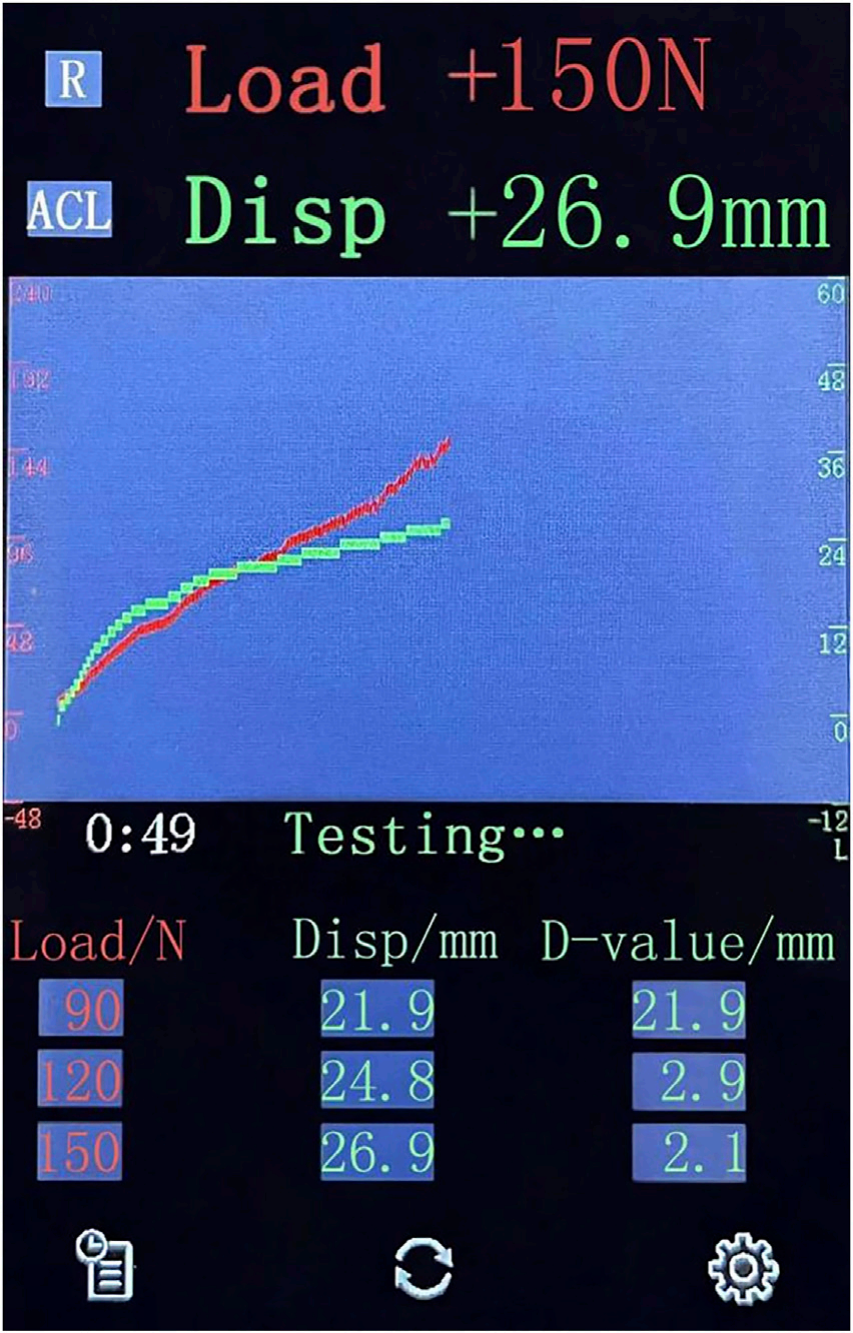
The display interface of the Ligs Digital Arthrometer for data collection. Load, the applied load; Disp, anterior displacement of the knee; D-value, the difference value of displacement between the current load and the prior load.

In the present study, the anterior knee laxity corresponding to the loads of 30, 60, 90, 120, and 150 N was automatically recorded and a force-displacement curve was generated (Figure 3). Each knee joint was measured three times, and the average of the three measurements was determined. After the measurement, the side-to-side difference (SSD) of anterior knee laxity at the corresponding load was calculated.

### 2.3 Data collection

The sex ratio, age, height, weight, BMI and SSD in anterior knee laxity at 30, 60, 90, 120, and 150 N loads were recorded for all subjects. Besides, we recorded the time from injury to measurement and the concomitant injuries of the medial collateral ligament (MCL), anterolateral ligament (ALL) and meniscuses for patients with complete ACL ruptures. A total of 246 subjects were enrolled in this study, which included 132 subjects with complete ACL ruptures in the experimental group and 114 normal subjects in the control group. The mean age of the subjects was 31.04 ± 5.35 years (range, 19 to 44 years) with a sex distribution of 171 males and 75 females.

### 2.4 Statistical analysis

A post hoc power analysis using G*Power software version 3.1.9.2 (Franz, Universitat Kiel, Germany) with a given effect size of 0.8 and α = 0.05 revealed a power of 1.

Data were analysed using the Statistical Package for Social Sciences (SPSS) version 26.0 (IBM Corp., Armonk, New York, United States). Categorical variables were expressed as frequencies and analysed using the chi-square test. Continuous variables were expressed as means and standard deviations. The normality of continuous variable distributions was analysed using the Shapiro‒Wilk test. If the distribution was normal, the parametric Student’s t-test was used for the quantitative variables. Otherwise, the non-parametric Mann‒Whitney *U* test was used. The optimal laxity thresholds of the Ligs Digital Arthrometer at 30, 60, 90, 120, and 150 N loads were determined according to receiver operating characteristic (ROC) curve analysis. The sensitivity (Se), specificity (Sp), positive predictive value (PPV), negative predictive value (NPV), positive likelihood ratio (LR+), negative likelihood ratio (LR-) and Youden’s index were calculated. Furthermore, the diagnostic value was evaluated by the area under the curve (AUC) with a 95% CI. Specifically, the diagnostic value corresponding to different AUCs can be divided into null (AUC = 0.5), poorly informative (0.5<AUC<0.7), fairly informative (0.7<AUC<0.9), highly informative (0.9<AUC<1), and perfect (AUC = 1) (Klouche, et al., 2015). A *p-*value <0.05 was considered statistically significant for all analyses.

## 3 Results

A total of 132 subjects, including 92 males and 40 females, were finally diagnosed with complete ACL ruptures by arthroscopy in the experimental group, and 46 cases were accompanied with MCL injuries (including 36 cases of grade I, 8 cases of grade II and 2 cases of grade III), 74 cases were accompanied with ALL abnormalities, 52 cases were accompanied with lateral meniscus injuries, and 46 cases were accompanied with medial meniscus injuries (including 7 cases of bucket handle tears). The mean time from injury to measurement was 8.69 ± 8.22 weeks and ranged from 1 to 42 weeks. The 114 normal subjects in the control group included 79 males and 35 females. The demographic data of the subjects were comparable between the two groups (Table 1). All the subjects were able to tolerate the maximum load of 150 N during the measurement.

**TABLE 1:** Demographic data of the subjects in two groups.

Variables	Experimental group	Control group	*p*-value
	(n = 132)	(n = 114)	
Sex ratio, male/female	92/40	79/35	0.946
Age, y	30.52 ± 4.94	31.65 ± 5.01	0.076
Height, cm	171.45 ± 6.24	170.34 ± 6.16	0.162
Weight, kg	68.78 ± 8.62	67.54 ± 11.37	0.332
Body mass index, kg/m^2^	23.39 ± 2.75	23.17 ± 3.03	0.550

*Data are given as the mean ± standard deviation or the number of subjects.

**FIGURE 4:**
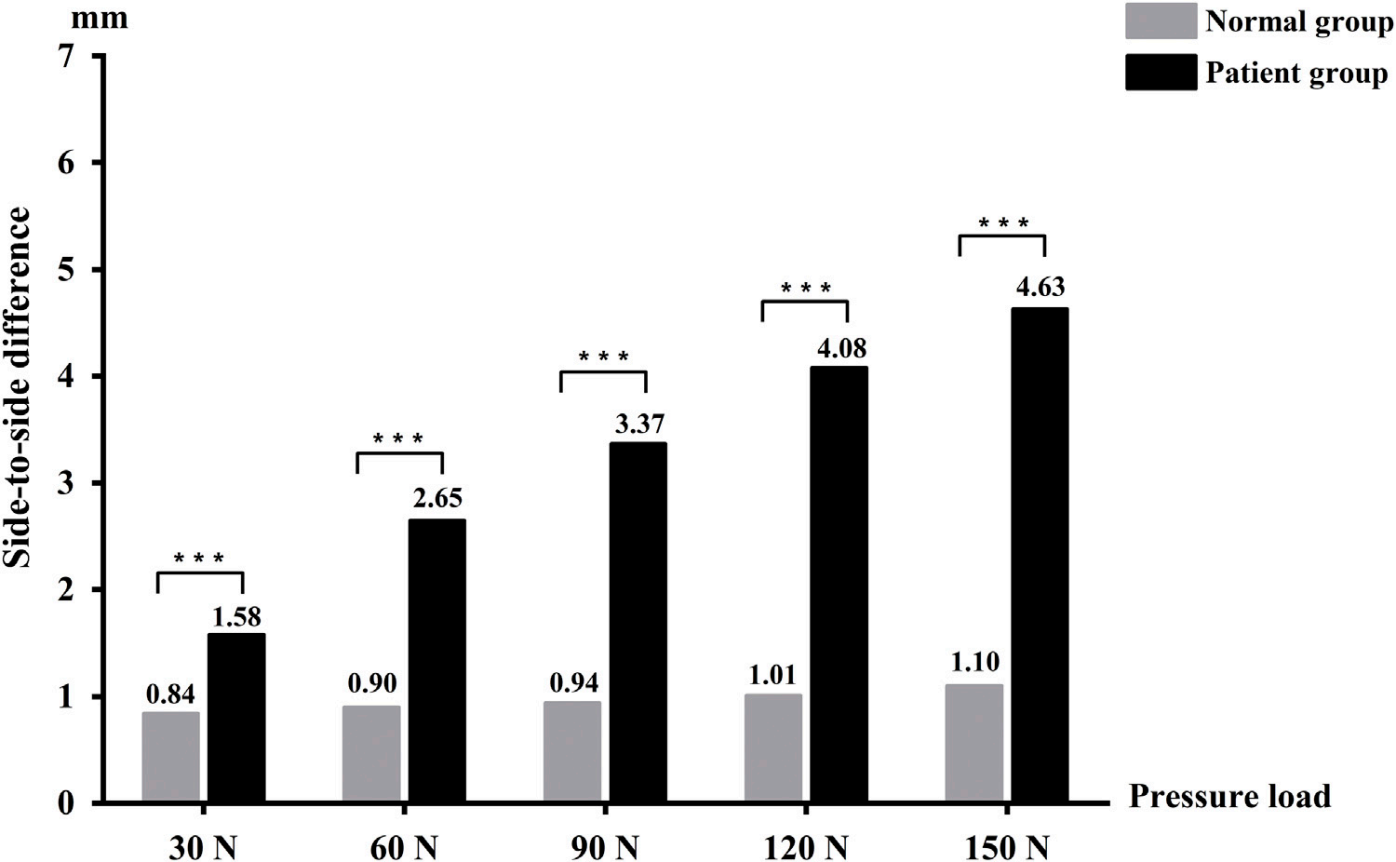
The side-to-side difference between the two groups in anterior knee laxity measured by the Ligs Digital Arthrometer.

The mean SSD of anterior knee laxity measured by the Ligs Digital Arthrometer at 30 N was 1.58 ± 1.07 mm in the experimental group and 0.84 ± 0.59 mm in the control group (*p* < 0.001). The mean SSD at 60 N was 2.65 ± 1.71 mm in the experimental group and 0.90 ± 0.67 mm in the control group (*p* < 0.001). At 90 N, the mean SSD was 3.37 ± 2.02 mm in the experimental group and 0.94 ± 0.70 mm in the control group (*p* < 0.001). At 120 N, the mean SSD was 4.08 ± 2.30 mm in the experimental group and 1.01 ± 0.71 mm in the control group (*p* < 0.001). At 150 N, the mean SSD was 4.63 ± 2.52 mm in the experimental group and 1.10 ± 0.79 mm in the control group (*p* < 0.001) (Figure 4).

According to the ROC curve analysis, the optimal laxity threshold for the diagnosis of complete ACL ruptures by the Ligs Digital Arthrometer was 1.1 mm SSD (Se = 66.7%, Sp = 69.3%) at 30 N, 1.3 mm (Se = 74.2%, Sp = 82.5%) at 60 N, 1.6 mm (Se = 79.5%, Sp = 94.7%) at 90 N, 1.9 mm (Se = 84.1%, Sp = 92.1%) at 120 N, and 2.1 mm (Se = 85.6%, Sp = 91.2%) at 150 N (Table 2).

**TABLE 2:** Results of the ROC curve analysis for the Ligs Digital Arthrometer.

Variables	30 N	60 N	90 N	120 N	150 N
SSD threshold, mm	1.1	1.3	1.6	1.9	2.1
Youden’s index	0.36	0.57	0.74	0.76	0.77
Se	66.7%	74.2%	79.5%	84.1%	85.6%
Sp	69.3%	82.5%	94.7%	92.1%	91.2%
PPV	0.68	0.78	0.93	0.93	0.92
NPV	0.64	0.72	0.81	0.83	0.85
LR+	2.17	4.24	15.00	10.65	9.73
LR−	0.48	0.31	0.22	0.17	0.16

SSD, side-to-side difference; Se, sensitivity; Sp, specificity; PPV, positive predictive value; NPV, negative predictive value; LR+, positive likelihood ratio; LR-, negative likelihood ratio.

**FIGURE 5:**
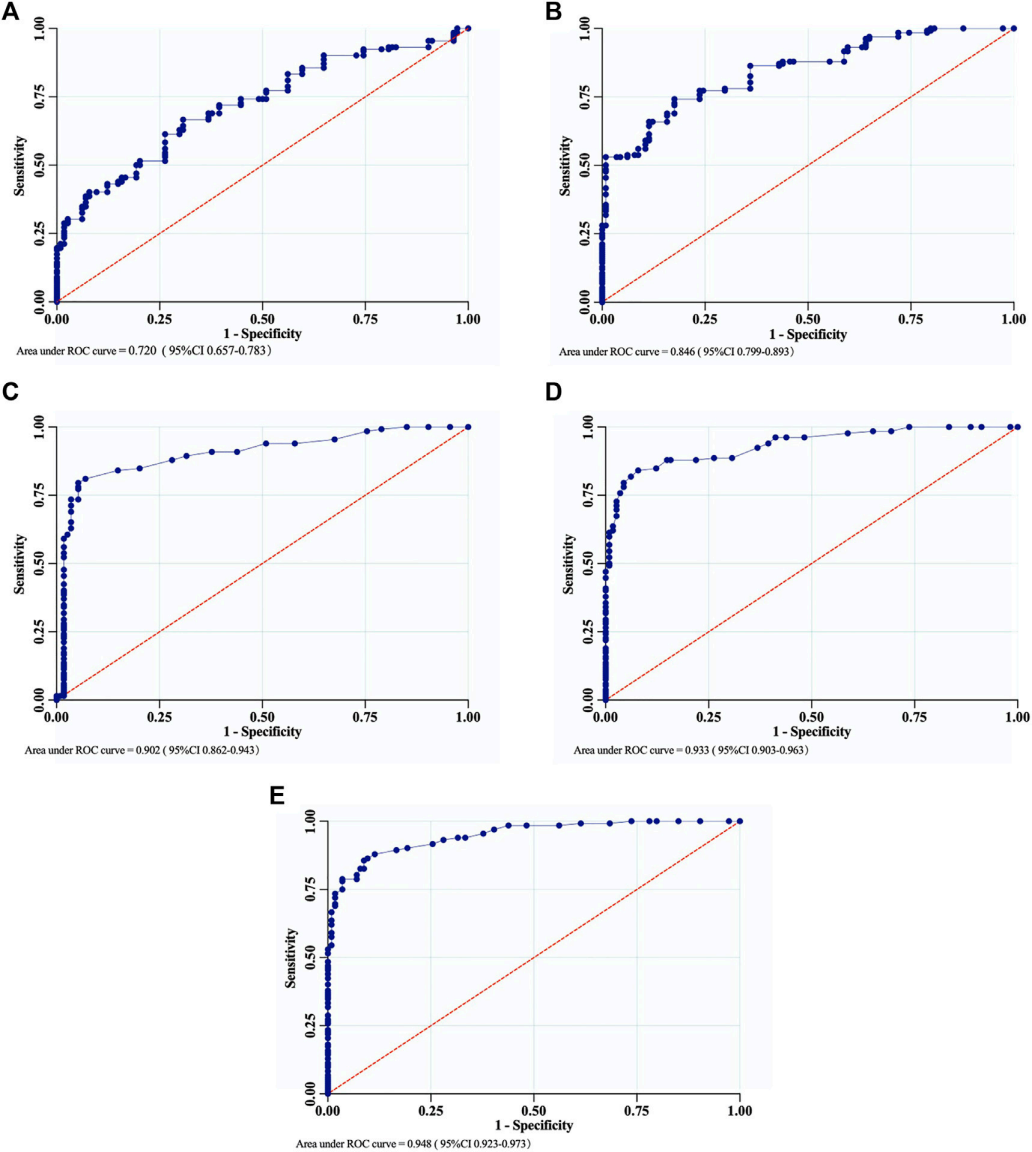
The ROC curves of the Ligs Digital Arthrometer for the diagnosis of complete ACL ruptures. **(A)** 30 N; **(B)** 60 N; **(C)** 90 N; **(D)** 120 N; **(E)** 150 N.

The AUC analysis showed that the measurements of the Ligs Digital Arthrometer were fairly informative at 30 N and 60 N loads, and highly informative at 90 N, 120 N, and 150 N loads. The AUC order at different loads from high to low was 150 N (0.948 [0.923–0.973])>120 N (0.933 [0.903–0.963])>90 N (0.902 [0.862–0.943])>60 N (0.846 [0.799–0.893])>30 N (0.720 [0.657–0.783]) (Figure 5).

## 4 Discussion

Clinical examination and MRI are the two main methods that are used to evaluate ACL ruptures (Kaeding et al., 2017). Although MRI is widely used as the preferred imaging method for the diagnosis of ACL ruptures with excellent sensitivity and specificity, it cannot be used to evaluate the severity of knee instability after injury (Koch et al., 2021; Shantanu et al., 2021). The anterior drawer test and Lachman test are commonly used physical examination to evaluate anterior knee laxity, but results are susceptible to patients’ pain, muscular tension and inherent knee laxity (Rohman and Macalena, 2016). In addition, the differences in clinician experience and applied manual force will lead to a certain degree of subjectivity in results and an inaccurate quantitative assessment of anterior knee laxity. In view of this problem, arthrometers have gradually become an important auxiliary device for the diagnosis of ACL ruptures and the quantitative evaluation of anterior knee laxity.

As a new versatile arthrometer, one advantage of the Ligs Digital Arthrometer is the ability to quantitatively assess the laxity of knee and ankle joints, by combining different fixation suites. A recent study by Chen et al. showed that the Ligs Digital Arthrometer is useful for quantifying the ankle laxity in chronic ankle instability with high diagnostic accuracy and excellent reliability (Chen et al., 2022). In terms of rehabilitation after ACLR, Zhou et al. assessed knee laxity after ACLR using the Ligs Digital Arthrometer to assist the guidance of return-to-sports decision-making (Zhou et al., 2022). Second, the Ligs Digital Arthrometer generates a force-displacement curve by delivering continuous loads and recording the laxity with a precision up to 0.1 mm, which allows the diagnosis of ACL ruptures based on the differential laxity of a force-displacement curve (Rohman and Macalena, 2016). Furthermore, another potential advantage of the Ligs Digital Arthrometer is that it can provide a specific load for simultaneous stress radiography. The imaging protocol of this approach is similar to that of the Telos device, which can directly evaluate the anterior tibial translation to avoid soft-tissue effects.

Therefore, for the diagnosis of ACL ruptures, the Ligs Digital Arthrometer can apply continuous loads to the patients, automatically record knee laxity with a higher accuracy on the screen, and provide a force-displacement curve when compared with the KT-1000 (Robert et al., 2009; Rohman and Macalena, 2016; Saravia et al., 2020). Although the GNRB can automatically measure knee laxity and has surface electrodes to control hamstring relaxation, the measurement is limited to the knee joint, and the instrument is not capable of realizing stress radiography like the Ligs Digital Arthrometer (Robert et al., 2009). In addition, the Telos device is specialized for stress radiography and cannot perform radiation-free arthrometry (Bouguennec et al., 2015).

The application of several arthrometers, such as the KT-1000, Genucom, Rolimeter, GNRB and the Stryker knee laxity tester, for the diagnosis of ACL ruptures has been previously reported (Mouton et al., 2016). The results of a meta-analysis showed that the sensitivity of the KT-1000 at 69 N was 54% for the diagnosis of ACL ruptures. The sensitivity and specificity of the KT-1000 were 78% and 92% at 89 N, respectively, while it had a better sensitivity of 93% and a better specificity of 93% at the maximum manual force. For the Genucom Knee Analysis System, the sensitivity was 74% and the specificity was 82%, while the Stryker Knee Laxity Tester had a sensitivity of 82% and a specificity of 90% (Van Eck, et al., 2013). Moreover, Panisset et al. found that the Rolimeter with SSD>5 mm had a 67.5% sensitivity and an 84.3% specificity for complete ACL ruptures (Panisset et al., 2012). For ACL-deficient knees, Ganko et al. found that the Rolimeter had a better sensitivity of 89% and a better specificity of 95% with SSD>3 mm diagnostic threshold (Ganko et al., 2000). Similarly, Passler et al. reported that the sensitivity and specificity of the Rolimeter for the diagnosis of ACL deficiency were 93% and 87%, respectively (Pässler et al., 1998) (Table 3).

**TABLE 3:** Results of the ROC curve analysis for different arthrometers.

Study	Arthrometer	Type of ACL rupture	Load	Se	Sp	Threshold
Van Eck et al. (2013) (meta-analysis)	KT-1000	Complete	69 N	54%	N/A	N/A
			89 N	78%	92%	N/A
			Maximum manual force	93%	93%	N/A
Van Eck et al. (2013) (meta-analysis)	Genucom	Complete	Maximum manual force	74%	82%	N/A
Van Eck et al. (2013) (meta-analysis)	Stryker	Complete	Maximum manual force	82%	90%	N/A
Panisset et al. (2012)	Rolimeter	Complete	Maximum manual force	67.5%	84.3%	5.0 mm
Ganko et al. (2000)	Rolimeter	ACL deficiency	Maximum manual force	89%	95%	3.0 mm
Pässler et al. (1998)	Rolimeter	ACL deficiency	Maximum manual force	93%	87%	N/A
Robert et al. (2009)	GNRB	Complete	134 N	70%	99%	3.0 mm
		Partial	134 N	80%	87%	1.5 mm
Klouche et al. (2015)	GNRB	Complete	89 N	92.2%	88.9%	1.0 mm
			134 N	92.2%	96.3%	1.5 mm
			200 N	92.2%	98.1%	1.9 mm
			250 N	90.6%	98.1%	2.1 mm
Saravia et al. (2020)	GNRB	Complete	134 N	90.7%	40.3%	3.0 mm
				74.4%	93.8%	6.8 mm
Beldame et al. (2012)	GNRB	Complete	89 N	48.6%	93.1%	1.5 mm
			134 N	59.4%	93.1%	
			150 N	59.4%	91.4%	
			250 N	62.2%	75.9%	
Lefevre et al. (2014)	GNRB	Partial	134 N	83.2%	64.3%	2.0 mm
			250 N	84%	81%	2.5 mm
Mouton et al. (2016)	GNRB	All types	200 N	75%	95%	1.2 mm

ACL, anterior cruciate ligament; Se, sensitivity; Sp, specificity; N/A, not available.

Furthermore, the validity of the GNRB for the diagnosis of ACL ruptures has been confirmed by several studies. Robert et al. found that the GNRB with SSD>3 mm had a sensitivity of 70% and a specificity of 99% at 134 N for the diagnosis of complete ACL ruptures, and at 134 N, it provided a sensitivity of 80% and a specificity of 87% with a 1.5 mm SSD diagnostic threshold in partial ACL ruptures (Robert, et al., 2009). A study by Klouche et al. reported the diagnostic value of the GNRB at different loads in complete ACL ruptures, and the GNRB with a 1.9 mm SSD threshold, had the highest diagnostic value at 200 N with a sensitivity of 92% and a specificity of 98% (Klouche, et al., 2015). Likewise, Saravia et al. found that the GNRB at 134 N delivered a sensitivity of 90.7% and a specificity of 40.3% with a threshold of 3 mm SSD for complete ACL ruptures. The threshold at 6.8 mm SSD offered a higher sensitivity (74.4%) and specificity (93.8%) with an AUC of 0.863 (Saravia, et al., 2020). Furthermore, Beldame et al. evaluated the validity of the GNRB in diagnosing complete ACL ruptures at loads of 89 N to 250 N (Beldame, et al., 2012). These results demonstrated that the GNRB with a 1.5 mm SSD threshold, had a sensitivity of 48.6% and specificity of 93.1% at 89 N, a sensitivity of 59.4% and specificity of 93.1% at 134 N, a sensitivity of 59.4% and specificity of 91.4% at 150 N, and a sensitivity of 62.2% and specificity of 75.9% at 250 N. For the diagnosis of partial ACL ruptures, Lefevre et al. found that the optimal SSD threshold of the GNRB was 2.5 mm with an AUC of 0.89, which had 84% sensitivity and 81% specificity (Lefevre, et al., 2014). Besides, Mouton et al. reported that the sensitivity and specificity of the GNRB at 200 N were 75% and 95%, respectively, with a threshold of 1.2 mm when diagnosing all ACL rupture types (Mouton et al., 2015) (Table 3).

To our knowledge, this study was the first to report the application of the Ligs Digital Arthrometer in the diagnosis of complete ACL ruptures. In the present study, the Ligs Digital Arthrometer provided a high diagnostic value at 90, 120, and 150 N loads. Simultaneously, we found that the diagnostic value improved with an increasing applied load in a certain range, which was similar to the results of previous studies (Highgenboten et al., 1992; Van Eck et al., 2013; Klouche, et al., 2015). Especially at 150 N load, the Ligs Digital Arthrometer delivered a better validity (Se 85.6% and Sp 91.2% at a 2.1 mm SSD threshold with an AUC of 0.95) for the diagnosis of complete ACL ruptures.

According to the findings of Lee et al., the diagnostic value of an arthrometer at the 30° knee flexion was significantly superior to that at the 45°, 60°, and 90° knee positions (Lee et al., 2019). Therefore, the 30° knee position, similar to the Lachman test, was used to measure anterior knee laxity by the Ligs Digital Arthrometer in the present study, which was helpful in increasing its diagnostic validity. During the measurement, the knee joint should be in neutral rotation because the anterior knee laxity measured in this state was considered as the intermediate displacement between the medial and lateral compartment (Papandreou, et al., 2005). Otherwise, the internal rotation of the knee joint decreased anterior knee laxity, while the external rotation resulted in an increase (Saravia, et al., 2020). Moreover, Ryu et al. found that meniscal tears or grade I MCL injuries accompanied by ACL ruptures may increase the laxity of the involved knee and improve the reliability of diagnostic tools (Ryu, et al., 2018). Besides, the method of SSD measurement was used to interpret the results to minimize the effect of inherent knee laxity and female hormones on the measurements of female subjects, which showed better consistency than absolute single-knee translation measurement (Rohman and Macalena, 2016).

Several limitations of the present study should be mentioned. First, we evaluated the validity of the Ligs Digital Arthrometer in complete ACL ruptures by only using the SSD values at specific loads, without further attempts to use the differential laxity of a force-displacement curve. Second, due to the small sample size of patients with partial ACL ruptures, no independent statistical analysis could be conducted to evaluate the validity of the Ligs Digital Arthrometer for diagnosing partial ACL ruptures. Moreover, further studies are needed to determine the reliability of the Ligs Digital Arthrometer in patients with ACL ruptures.

## 5 Conclusion

The Ligs Digital Arthrometer proved to be of high diagnostic value in complete ACL ruptures at 90 N, 120 N, and 150 N loads. The diagnostic value improved with increasing load in a certain range. Based on the results of this study, as a portable, digital and versatile new arthrometer, the Ligs Digital Arthrometer was a valid and promising tool for diagnosing complete ACL ruptures.

## Data Availability

The raw data supporting the conclusion of this article will be made available by the authors, without undue reservation.
